# An Overview on Different L-Thyroxine (l-T_4_) Formulations and Factors Potentially Influencing the Treatment of Congenital Hypothyroidism During the First 3 Years of Life

**DOI:** 10.3389/fendo.2022.859487

**Published:** 2022-06-09

**Authors:** Stefano Stagi, Giovanna Municchi, Marta Ferrari, Malgorzata Gabriela Wasniewska

**Affiliations:** ^1^ Department of Health Sciences, University of Florence, Anna Meyer Children’s University Hospital, Florence, Italy; ^2^ Department of Human Pathology of Adulthood and Childhood, University of Messina, Messina, Italy

**Keywords:** congenital hypothyroidism, L-thyroxine (L-T4), central nervous system, children, plastic period, pharmacological interferences

## Abstract

Congenital hypothyroidism (CH) is a relatively frequent congenital endocrine disorder, caused by defective production of thyroid hormones (THs) at birth. Because THs are essential for the development of normal neuronal networks, CH is also a common preventable cause of irreversible intellectual disability (ID) in children. Prolonged hypothyroidism, particularly during the THs-dependent processes of brain development in the first years of life, due to delays in diagnosis, inadequate timing and dosing of levothyroxine (l-thyroxine or l-T_4_), the non-compliance of families, incorrect follow-up and the interference of foods, drugs and medications affecting the absorption of l-T_4_, may be responsible for more severe ID. In this review we evaluate the main factors influencing levels of THs and the absorption of l-T_4_ in order to provide a practical guide, based on the existing literature, to allow optimal follow-up for these patients.

## 1 Introduction

Congenital hypothyroidism (CH) is a relatively frequent congenital endocrine disorder, due to the defective production of thyroid hormones (THs) at birth (1, 2). CH most commonly occurs because of disruption of thyroid gland development or a TH biosynthesis disorder (primary hypothyroidism); it can also be related to a defect in the release of thyroid-stimulating hormone (TSH), a condition which rarely occurs in isolation and is commonly associated with the deficiency of other pituitary hormones (combined pituitary hormone deficiency or CPHD) (secondary or central congenital hypothyroidism or cCH) (1, 2).

CH is also one of the most common preventable causes of irreversible intellectual disability (ID) in children (3) but early diagnosis and treatment can prevent serious consequences (4). THs are involved in the development of normal neuronal networks, and if CH is not treated it can lead to severe, permanent alterations in brain anatomy and function (5). It is important to consider that many complications, such as brain disorders and injuries and nerve growth retardation, may not be clinically recognizable in the first weeks of life leading to delay in the introduction of treatments that can prevent disabling and severe outcomes (4, 6, 7).

Moreover, during pregnancy, absent or inadequate maternal levothyroxine (l-thyroxine or l-T_4_) treatment correlates to an increased risk of obstetric complications at birth, as well as neuropsychological problems in the unborn child (8), adding to damage associated with neonatal CH.

Screening programs for CH are in place in many countries, allowing for the prompt initiation of treatment during the first months of life, frequently before clinical signs and symptoms appear (1). Generally, the recommended period for collecting blood samples for thyroid screening is between the 3^rd^ and 5^th^ day of life (9). The detection of a suspected thyroid dysfunction should be confirmed with secondary tests, which should be carried out promptly, ideally within the second week of life. However, despite the clear benefits of screening programmes, 70% of infants worldwide are born in areas without routine neonatal screening (10).

In CH, the incidence rate is reported to be about one in 2,000 - 4,000 live births, with a female-to-male ratio of nearly 2:1, although there are significant differences between countries (1–3): for example, in the United States, the incidence is 1 in 3500 to 5000, in Europe 1 in 3000 and in Japan 1 in 5700 (1–3). These differences are due to a number of factors including ethnicity, environmental influences, conditions affecting birth and pregnancy, and the presence or absence of screening programs (1–3). Moreover, sexual dimorphism can affect prenatal thyroid migration (11). The number of low birth weight (LBW) infants, who have a higher risk of CH, has increased in recent years due to improvements in neonatal intensive care (12).

Approximately two thirds of CH cases are secondary to thyroid dysgenesis which can involve both Mendelian and non-Mendelian inheritance: in fact, although CH is typically sporadic, familial cases have been described (1–3). In patients with a genetic form of CH, germline mutations in genes involved in thyroid gland organogenesis (i.e. *PAX8*, *TTF-1/NKX2.1*, *TTF-2/FOXE1* and *TSHR*) and responsible for thyroid dysgenesis are rarely found (1–3). Some cases of CH may result from inborn errors in the synthesis of THs (1–3). The clinical expression of mutations in some genes involved in disorders of thyroid function, such as *DUOX or DUOX2/DUOXA2*, may vary widely between individuals and over time; some patients who present thyroid function impairment in the first weeks of life do not require treatment (13). Initial l-T_4_ doses and follow-up should be adapted to the needs of each CH patient taking into consideration the formulation used and THs levels.

Practices governing CH diagnosis and treatment have undergone significant changes in recent years: treatment with higher starting doses of l-T_4_ is now the treatment of choice which has led to improvements in both auxological (14–16) and neurological prognoses (17, 18).

In all cases, there is a significant increase in TSH and/or a significant reduction in FT_4_. Treatment should be promptly initiated with careful follow-up to determine appropriate dosage (13). Many guidelines (13, 19–21) for treating CH suggest a higher initial l-T_4_ dose (generally at least 10-15 μg/kg/day) than the 5-10 μg/kg/day previously recommended, in order to normalize thyroid function more quickly (optimally within the first 14 days of treatment) and achieve better development quotient (DQ) scores. It appears that bone maturation at birth may predict psychomotor development in the first 12 months of life (22).

Several studies (13, 23–26) on the neurological outcomes of CH patients confirm that a rapid normalization of thyroid function can reduce the incidence of neurological impairment (19).

However, one study noted, for the first time, that children treated with high doses of l-T_4_ were more likely to present hyperactivity, aggression and delinquency (27). Other data have also revealed that high serum T_4_ levels may be associated with reduced attention in children of school age (28) and that there may exist a relationship between permanent attention deficit hyperactivity disorder (ADHD) and overtreatment in early years of life (29). These data show that overtreatment of CH can be dangerous and stress the importance of accurate follow-up to monitor dosage of l-T_4_.

## 2 Treatment and Monitoring of Primary CH

### 2.1 Goals of Treatment for CH

Triiodithyronine (T_3_) is the biologically active form of TH but it is important to remember that as T_3_ cannot pass the brain barriers in significant quantities, most T_3_ in the brain is converted from local deiodination of T_4_, meaning that in CH patients, it is not necessary to replace T_3_ to normalise neurologic development (1). Research involving 47 infants treated with different doses of l-T_4_, found that serum T_3_ normalized and stayed normal regardless of dosage, indicating that l-T_4_ alone is an adequate treatment for CH (30).

It is important to start treatment promptly in all infants who test positive in CH screening, after samples for confirmatory tests are taken, the results of which will determine the optimal l-T_4_ starting dose (31). When the results of venous blood tests are not available on the same day, treatment should be started without waiting for the results, particularly if the patient’s condition is a cause for concern (30, 32, 33),

The treatment goals for CH are outlined by the American Academy of Pediatrics (AAP) (30) and by the European Society for Paediatric Endocrinology (ESPE) (20) and can be summarized in the following main points:

- keeping serum Free T_4_ (FT_4_) above the average age-adjusted normal level but within the upper limit, especially in the first 12 months of life.
- keeping serum TSH below 5 mU/L.

Some data suggests that infants with serum T_4_ levels under 10 μg/dL during the first 12 months of life, associated with serum TSH levels greater than 15 mU/L, have a lower Intelligence Quotient (IQ) than those with serum T_4_ levels above 10 μg/dL (28). Furthermore, other data show that treatment with higher doses of l-T_4_ may be connected with higher IQ at 7 and 8 years of life, particularly in verbal working memory and sentence comprehension (27). Frequent and significant alterations in serum T_4_ and TSH levels during the first 12 months of life are associated with differences in mental development index scores and verbal IQ (27, 33).

Prompt diagnosis and initiation of therapy for CH is essential for ensuring positive outcomes, especially in high-risk infants (34). However, some patients with CH may not achieve normal TSH concentrations despite l-T_4_ treatment even at higher doses. Poor adherence to treatment is the most frequent reason for poor TSH control. Therefore, in patients who do not respond to treatment it is important to investigate whether l-T_4_ is being correctly administered and if necessary, review the method of administration. The possibility of impaired l-T_4_ absorption related to gastrointestinal diseases or the interference of drugs or other substances (see below) should also be considered (35).

Good therapeutic management practices appear to be of particular importance to ensure optimal results in terms of neuromotor and mental development, due to the THs-dependent processes of brain development in the first years of life. Professionals should provide clear explanations and instructions to caregivers to improve adherence to treatment (9, 34, 35). It is critical that families comprehend that treatment is essential for the best patient outcomes.

### 2.2 Dosage and Timing

Infants with severe forms of CH are at a higher risk for ID; for this reason, the dosage and timing of l-T_4_ treatment are fundamental for optimising neurocognitive outcomes in the future. Delays in normalizing serum T_4_ of more than one week can lead to lower IQ (23). Some data suggest that scores in behavioural and cognitive tests were lower for patients whose T_4_ did not normalize before two weeks than for patients who achieved normal levels in under two weeks (24). Therefore, as stated above, the treatment goal is to bring serum T_4_ to above below 129 mmol/L (> 10 μg/dL) as quickly as possible. The data available in the literature show that there is an inverse relationship between the initial l-T_4_ dose, and the time taken to reach target serum T_4_ levels (24) meaning that the prompt administration of an appropriate starting dose of l-T_4_ is particularly important.

The starting l-T_4_ dose recommended both by AAP and the ESPE is from 10 to 15 µg/kg/day (19–21, 30). In infants born at term this translates to an average starting dose of 37.5 - 50 µg per day (20, 21). In the above cited research, infants receiving 50 µg per day (corresponding to 12 - 17 µg/kg/day), versus 37.5 µg (corresponding to 10 - 15 µg/kg/day) performed better in tests measuring behaviour, reading, spelling and maths. The patients on the higher dose achieved IQ scores 11 points higher than those on the lower dose (24). Therefore, in infants whose serum T_4_ levels are lower than 5 μg/dL, higher l-T_4_ doses are recommended.

The goal is to normalize T_4_ and TSH within 2 and 4 weeks, respectively (19, 24). T_4_ and FT_4_ concentrations should be in the upper half of the reference range for age, with normalization of TSH (19).

These positive outcomes associated with administering higher doses of l-T_4_ have been highlighted by studies in Europe, the United States and Canada (24, 36–38). In one study, 83 infants were placed into three different groups, each receiving different initial doses of l-T_4_ from diagnosis at birth (the first group was treated with 6.0 - 8.0 µg/kg/day, the second with 8.1 - 10.0 µg/kg/day, and the third with 10.1 - 15.0 µg/kg/day) and followed up at four years of age for growth and intellectual development. The infants with severe CH who started on the highest dose achieved the highest intellectual scores (39). Another study involving 61 infants comparing early treatment with delayed treatment and low doses with high doses, found that the outcomes for patients with severe forms of CH were more favourable in those treated early (under 2 weeks) and with higher doses (>9.5 µg/kg/day). Such patients presented normal psychomotor development at 10-30 months of age (23), showing that the time it takes for TSH to normalize is inversely correlated to neurological outcome (19).

Another study which analysed data on the intellectual development of 45 CH children found that differences and variations in serum levels of T_4_ and TSH during the first year of treatment predicted scores on mental development index (MDI) and verbal IQ tests performed at 2 years and 6 years of age (33). It is clearly imperative to monitor patients so that l-T_4_ doses can be promptly adjusted and optimal T_4_ and TSH levels maintained.

### 2.3 Recommended Follow up During the First Three Years of Life

CH treatment must be carefully monitored with appropriate adjustment of the l-T_4_ dose, until serum TSH and FT_4_ concentrations have normalized. Clinical evaluations must be made every few months during the first three years of life together with frequent measurements of serum T_4_ or FT_4_ and TSH, in order to avoid prolonged periods of under- or overtreatment (19, 24). Management should focus on maintaining a status of euthyroidism, especially in the first 3 years of life when brain development is most dependent on THs. However, in CPHD patients with associated adrenal insufficiency or in patients in whom this problem cannot be excluded, adrenal function should be investigated, and glucocorticoid therapy should precede l-T_4_, replacement treatment to avoid inducing an adrenal crisis (19).

The AAP recommends monitoring thyroid function 2 and 4 weeks after initiating l-T_4_ treatment and every 1 to 2 months during the first 6 months of age. After this, patients should be monitored every 3-4 months between 6 months and three years of age, and then every 6-12 months until the child reaches adult height. Moreover, TH should be evaluated four weeks after any change in dose and follow-up evaluations need to be more frequent if results are abnormal or non-adherence to therapy is suspected (19).

Although in most infants, serum T_4_ will normalize within seven to fourteen days and serum TSH will normalize within one month of treatment, some patients continue to present altered TSH levels (10-20 mU/L) or serum T_4_ levels beyond these timeframes (40). This is often caused by under treatment, although it is important to remember that in 10% of CH infants undergoing treatment the FT_4_ feedback control on TSH secretion does not mature normally (41) due to the resetting of the pituitary-thyroid feedback mechanism *in utero* (42). In one paper reporting data for 42 CH paediatric patients, a prevalence of pituitary TH resistance of 43% in patients below one year of age was found. However, in childhood and adolescence this problem was found in only 10% of patients (42), suggesting that TH resistance is more common in younger patients and may decrease with age.

### 2.4 Effects of Inadequate or Non-Compliance

The availability of adequate TH is essential for normal brain development in the first two to three years of life. Low TH levels during this period can lead to irreversible damage, whereas after 3 years of age the effects of hypothyroidism can usually be reversed when the condition is treated. A study by the New England Congenital Hypothyroidism Collaborative highlighted the importance of regular monitoring and dose adjustments to control serum FT_4_ and T_4_ levels. Researchers found that eighteen infants included in the study with reduced serum T_4_ levels, who were taking insufficient doses of l-T_4_ (< 5 µg/kg/day) and who had an anamnesis showing poor compliance in the first three years of life, had a mean IQ of 87 (43). In comparison a larger group of correctly treated patients, with a history of normal serum T_4_ levels, achieved a mean IQ score of 105.

Further data suggest that noncompliance after the first three years of life may also negatively affect cognitive achievement. For example, researchers made unannounced home visits on 14-year-old adolescents with CH and found that 44% were not adequately treated, having serum TSH > 15 mU/L and T_4_ levels < 6.6 µg/dL in the majority (44). Psychometric tests revealed a mean IQ of 106. The importance of complying with TH treatment was explained to patients and their families (the dose was not altered), and 12 to 24 months later, serum TH levels had improved, and the repetition of psychometric tests showed an improvement in mean IQ of 6 points. Although the study did not include a control group, the authors concluded that non-compliance during adolescence is frequent, and that improved compliance can lead to improved cognitive function.

ID caused by CH is preventable but lack of awareness amongst caregivers about the benefits of treatment may be a barrier to clinical improvement in some patients (7). Newborn screening represents a unique opportunity for avoiding damage connected to CH (7).

### 2.5 Effect of l-T_4_ Overtreatment

The effects of overtreatment with l-T_4_ are well known (45–47). A prospective study by Rovet et al. (27) demonstrated for the first time that, despite an improvement in cognitive profiles, an initial high dose of l-T_4_ can be associated with behavioural problems in childhood. Bongers-Schokking et al. (48) evaluated DQ scores at 11 years, showing that overtreatment can lead to a cognitive impairment of −17.8 points if it is prolonged and −13.4 points when the overtreatment is limited to the first 2 years of life. Another recent study by Bongers-Schokking et al. (29) concluded that episodes of overtreatment during the first 3 months can lead to permanent ADHD, while undertreatment may be related to autism.

## 3 Treatment and Monitoring of cCH

Many data and recently updated CH consensus guidelines support the treatment of cCH with a once daily administration of l-T_4_ (13). In severe cCH (serum FT_4_ levels before treatment below 5 pmol/L), the minimum l-T_4_ starting dose should be no less than 10 μg/kg/day, in order to promptly bring FT_4_ within the normal age-adjusted range (49). A lower starting dose of l-T_4_ (from 5 to 10 μg/kg per day) should be used in milder forms to avoid overtreatment. For primary CH, the long-term biochemical aim must be to bring and maintain serum FT_4_ within the upper half of the reference interval for the patient’s age (49). Although it remains essential to conduct randomized clinical trials in children to obtain specific results for the paediatric population, data from adult studies support this approach (50, 51).

cCH should not be monitored by measuring serum TSH. Instead, serum FT_4_ is the most reliable indicator of efficacy of treatment. If TSH levels prior to treatment are low, it is not necessary to take subsequent TSH measurements (13, 49).

Clinical and biochemical follow-up for patients with cCH should be similar to that for patients with primary CH. After diagnosis and the start of l-T_4_ treatment, a first evaluation should be carried out within 1-2 weeks. If testing indicates a good response, subsequent evaluations should be scheduled every two weeks until serum FT_4_ normalizes. After this phase, patients should be evaluated every 1-3 months during the first year of life, every 2-4 months during the successive two years, and then every 3-6 months. When it is necessary to take blood samples to measure serum FT_4_, parents or caregivers must be instructed not to administer the daily dose of l-T_4_ beforehand. If this is not possible, blood should be taken at least four hours after the last l-T_4_ dose (13). For cases in which under- or overtreatment is suspected, it may be useful to measure TSH, and free or total T_3_. Patients with FT_4_ near the lower limit of the age-adjusted range should be considered at risk for undertreatment, especially if TSH >1.0 mIU/L while those with FT_4_ near or above the upper limit of the age-adjusted range should be considered at risk for overtreatment, especially if there are typical clinical signs of thyrotoxicosis or high FT_3_ levels (52).

Naafs et al. found that 86% of 92 Dutch children with cCH detected by newborn screening had both CPHD and ACTH deficiency and that 96% and 74% had GH and gonadotropins deficiency respectively (53). Most of the cases of ACTH deficiency (71%) were diagnosed in the first month of life, but GH deficiency on average was not diagnosed until 1.3 years of age. These findings demonstrate the importance of monitoring all children with cCH over time for the possible emergence of other pituitary defects. Naafs JC et al. (54) also reviewed studies on children with cCH present in literature and found that the full-scale intelligence quotient was below 85 in 27% of patients, and below 70 in 10%. However, the age treatment was begun was not known for most patients (54).

## 4 Pharmacokinetics and Different Formulations of l-T_4_

The pharmaceutical preparation of drugs and the mode of administration are important elements in achieving and maintaining euthyroidism in CH and counteracting the effects of external influences on THs values.

Synthetic compounds of thyroxine usually contain the *laevo* isomer of thyroxine (l-T_4_), most commonly as a sodium salt (55); however, although l-T_4_ is frequently prescribed worldwide, the correct daily dosage, mode of administration, and approach to resolving treatment failure remain matters of debate. In primary CH, in order to maintain euthyroidism, thyroxine must be efficiently absorbed and unbiased serum TSH values must be available, which cannot be measured in patients with cCH.

Physiologically, l-T_4_ is absorbed in the small intestine and, to a lesser extent, in the stomach (56). In patients with short bowel syndrome, the absorption of l-T_4_ can be compromised (57). Although the pharmacokinetic parameters of l-T_4_ have not been assessed in children, in adults there are differences between euthyroid and hypothyroid subjects. Hypothyroid subjects achieve C_max_ with a delay of 2-3h, associated with a decreased volume of distribution, and a higher bioavailability (than the standard 60–80%) (57–59).

l-T_4_ permeation through the intestinal epithelium occurs principally in the duodenum and the jejunum, largely within 3 h after ingestion; absorption is most rapid within the first 2 h (57). Approximately 70% of the l-T_4_ contained in tablets is absorbed and l-T_4_ ingestion is best in the morning, at least 1 h before breakfast as fasting improves absorption (60).

Concomitant gastrointestinal diseases, such as coeliac disease (CD), *Helicobacter pylori* infection, lactose intolerance, inflammatory bowel diseases, as well as parasitic infestation (*Giardia lamblia*) may cause l-T_4_ malabsorption (56, 57).

Increased gastric pH is also known to influence l-T_4_ absorption (61); many studies confirm that individuals with impaired gastric acid secretion, as a result of disease or the use of proton pump inhibitors (PPI), often require higher l-T_4_ doses to reach target TSH levels (62). In comparison, the available data suggest that pantoprazole and esomeprazole do not alter the pharmacokinetic parameters of l-T_4_ absorption, although the evidence is insufficient for this to be certain (63).

Generally, the half-life of l-T_4_ in a state of euthyroidism is 6-7 days (3-4 days with hyperthyroidism and 9-10 days with hypothyroidism). The full therapeutic effect of thyroxine is evident 3-4 weeks after treatment is initiated; if treatment is interrupted the therapeutic effect continues to be observed for 1-3 weeks. Because thyroxine has a long half-life, dose changes should not be made any more frequently than every 3-4 weeks (55).

Some studies also suggest that changing the drug’s formulation can significantly reduce problems of l-T_4_ malabsorption. Liquid l-T_4_ is absorbed faster than solid pills (64), and has also been found to be less affected by gastric pH and conditions which impair l-T_4_ absorption (61, 65). Liquid l-T_4_ seems more effective in reducing TSH levels than tablets of the same dose, independently of whether malabsorption problems are present (65, 66). In patients assuming PPI or other interfering drugs, switching from tablets to a liquid formulation may improve the efficacy of treatment (67, 68). l-T_4_ in soft gel capsules also dissolves better in increased pH (69) than tablets and appears able to reduce TSH levels both in patients with gastro-intestinal diseases and in patients without apparent malabsorption problems (70, 71).

Serum TSH is not always a reliable marker for pharmacological euthyroidism since values are affected by several drugs and some pathophysiological conditions (72, 73). However, it remains the best available marker as it is not easy to directly measure thyroxine absorption. The daily dose of l-T_4_ required to obtain the desired TSH value/therapeutic effect does not directly correlate to the ingested dose as absorption of oral l-T_4_ is between 60–80 %. Often high l-T_4_ doses are necessary to attain target serum TSH concentrations, which can lead to costly hormonal monitoring. Repeated failure in reaching therapeutic targets is common. Dosage often has to be modified in children as they grow and gain weight, especially in the first year of life, although the need for higher does may also be due to impaired l-T_4_ absorption.

It is important to consider the time of day that the drug is taken. Tablets should be taken at least 1 h before breakfast (60). In cases of reduced absorption or in patients with CD, lactose intolerance or other chronic inflammatory conditions, liquid l-T_4_, or soft gel capsules may be better absorbed.

### 4.1 Different l-T_4_ Formulations

l-T_4_ is available in several forms (tablets, soft gel capsules, liquid and, in some countries, powder for preparing an intravenous solution). Unfortunately, tablets are the only form available in many countries.

#### 4.1.1 Tablets

l-T_4_ tablets are the oldest treatment for CH. l-T_4_ is absorbed within 20–30 min after ingestion and the absorption process takes about 3 h (59). Taking tablets with food affects the drug’s pharmacokinetics and the efficacy of treatment; t_max_ is delayed, and the peak value of l-T_4_ absorption decreases (57, 74), as does drug bioavailability, from 15% to as much as 40% (75, 76). Not refraining from eating for at least 30 min after l-T_4_ tablet ingestion, may result in significantly higher TSH levels (77). This is important to bear in mind in patients for whom persistent or frequent TSH alterations can be dangerous such as congenital hypothyroid patients, pregnant women, patients with cardiac disease, or oncological patients (78).

For young infants, the tablet should be crushed and mixed with breast milk or water. It should not be mixed with soy formula which interferes with absorption. If the infant requires soy formula, l-T_4_ should be given midway between feeds and thyroid function must be carefully monitored (79).

Although stable in dry air, l-T_4_ becomes very unstable in the presence of light, heat and humidity. It is important to make sure that parents and other caregivers store the drug in its original container, away from sunlight and in a cool, dry place (55). Incorrect storage can affect bioavailability, making absorption more difficult which can cause fluctuations in the levels of THs.

#### 4.1.2 Liquid Form

A liquid solution of l-T_4_ containing ethanol is available in some countries. This form contains l-T_4_, ethanol, and glycerine and appears to be more stable than tablets. Its main advantage is that the gastric phase of digestion is not necessary (64), meaning that it is absorbed more quickly (64) as has been confirmed by *in vitro* (80) and *in vivo* (81–84) studies conducted in hypothyroid adults assuming l-T_4_ while fasting or with breakfast (81, 84). The faster onset of absorption, compared to tablets, minimizes the risk of drug-food interactions (64).

Studies conducted on paediatric patients affected by CH showed that the liquid formulation is a safe alternative to tablets, ensuring normal growth and neuromotor development (85–87). However, there appears to be greater suppression of serum TSH during the first months of treatment, indicating a higher risk of overtreatment (85–87).

Another concern about the liquid formulation is the potential risk of long-term side effects from the chronic administration of excipient ethanol; however, one millilitre of liquid formulation, equal to 100 μg of l-T_4_, contains 243 mg of ethanol as excipient, more than four times lower than the safe threshold for alcohol established by the AAP (88). Several studies on treatment during pregnancy or lactation do not demonstrate any side effect on infants (89–91).

A recent multicentric Italian study involving 253 CH patients, with a follow-up period of up to 3 years, confirmed the efficacy of l-T_4_ at high doses (87) in both liquid and tablet form. The data confirmed findings of previous studies by Cassio et al. and Peroni et al. (85, 86) and are in line with the results of a meta-analysis conducted by Aleksander et al., which showed that a high initial l-T_4_ dose of between 10 and 15 μg/kg/day was effective and safe in all CH patients and that high FT_4_ values during infancy did not correlate with IQ impairment at adolescence (18).

In a study by Vigone et al. patients treated with oral l-T_4_ solution presented, after 15 days and 1 month, but not at 3 months, significantly lower median TSH and higher FT_4_ values than those treated with tablets, with suppressed TSH levels in about 60% of the CH patients, indicating a higher risk of overtreatment during the first month of therapy (87). This may be due to a higher absorption of liquid l-T_4_ suggesting that to avoid overtreatment, lower starting doses should be administered with shorter intervals between follow up visits (87). Both tablet and liquid l-T_4_ were associated with adequate total DQ scores at 1 and 3 years of follow-up (85–87).

Another, prospective randomized control study in children with CH, to assess the efficacy and safety of liquid l-T_4_ in comparison to tablets, evaluated thirty-nine children, aged 3-12 years old. At six months the group treated with liquid l-T_4_ had higher TSH levels than the group treated with tablets, while FT_4_ levels had no statistical difference. Dose adjustments were more frequent in the group treated with liquid. l-T_4_ (92).

Finally, recently, another liquid formulation containing l-T_4_, but without ethanol, using a calibrated oral syringe, has been studied, even if only in extremely premature infants (born below 28 weeks’ gestation), without apparent effect on brain size (93).

#### 4.1.3 Soft Gel Capsule

In this form, l-T_4_ is dissolved in water and glycerine, and conserved in a gelatine matrix, to protect the hormone from degradation. It is free from lactose, gluten, alcohol, sugar, and dyes (94). The literature does not report studies in children with CH. One study, conducted in 60 euthyroid adults, investigated the effect of meals on the absorption of l-T_4_ in this form. Patients who had been taking oral liquid l-T_4_ with breakfast, switched to the soft gel capsule, without changing the mean dose. TSH, FT_4_, and FT_3_ levels were measured at the beginning of treatment with the soft gel capsule and after 6 months (95). There were no significant differences in TSH, whereas FT_4_ and FT_3_ levels were significantly lower in the subjects treated with the soft gel capsule (95). Another study found that, unlike l-T_4_ tablets, the absorption of soft gel capsules is not affected by the concurrent consumption of coffee (96), suggesting that capsules may be of benefit for patients with CD, lactose intolerance and other chronic inflammatory conditions. A paper, evaluating these new formulations of oral l-T_4_ for adults with central hypothyroidism, reported that liquid or soft gel l-T_4_ was more effective at bringing serum FT_4_ levels into the upper-normal range (97).

## 5 Effects of Foods, Drugs/Diseases, Supplementation, and Nutrition Route on THs Levels

Many physiological, nutritional, pharmacological or pathological factors can impair the intestinal absorption of oral l-T_4_ (57, 98, 99). Patients, parents and carers often underestimate the risks of eating concomitantly or too soon after taking l-T_4_ which can negatively affect absorption and therefore the efficacy of the therapy (100).

This is particularly worrying for paediatric CH patients, especially in the first three years of life given the thyroid-dependence of the central nervous system during this phase. Parents need to be made aware of the importance of correctly administrating l-T_4_ to avoid interference from food and should be reminded to inform their child’s paediatrician and endocrinologist about any drugs or medicaments the patient has taken or is taking.

Many drugs or medications can alter TH levels by affecting the hypothalamic and pituitary regulation of thyroid hormone production, the synthesis and secretion from the thyroid gland directly, the metabolism of THs (deiodination, sulfation and glucuronidation) or by altering affinity for levels of thyroxine binding globulin (100).

A number of drugs, such as proton-pump inhibitors, sucralfate and aluminium-containing antacids, affect the absorption of l-T_4_ by changing gastric pH and reducing the solubility of tablets. Others, such as iron and calcium salts, phosphate binders, bile acid sequestrants and other resins, bind with l-T_4_ to form insoluble complexes. The mechanism of interference for some drugs, such as raloxifene, is unknown (99). Certain herbal remedies also impair the ability to absorb l-T_4_ (101) and nutritional supplements can also interfere, including infant formulas containing soy protein and iron, calcium and fibre supplements (100).

It is important to inform parents and caregivers about the possible interference of drugs and supplements and the potential damage they can cause in patients who are dependent on exogenous l-T_4_ (100). The need to increase the dose of l-T_4_ does not always signal gastrointestinal malabsorption but may indicate interference from food or medications (98). For this reason it is essential to take a patient’s diet and medication use into consideration.

In a study of 925 adult hypothyroid patients aim at identifying factors with an effect on l-T_4_ therapy, McMillan et al. found that over 50% assumed dietary supplements with a potential interaction with l-T_4,_ such as calcium (over 47% of cases) and iron (almost 12% of cases), while 68% reported frequent intake of foods rich in fibre, iodine, or soy (102). A study by Michel et al. (103) also found that many patients had insufficient awareness of the importance of l-T_4_ administration schedules.

The problem is also of concern in paediatric patients, as dietary supplements are often given to infants and children (104). Some data suggests that more than 20-30% of children take dietary supplements regularly, most of which have not been recommended by a health care provider (104).

### 5.1 l-T_4_ – Food Interaction

It is clear that interference from food can negatively impact the efficacy, and in some cases the safety, of treatment with l-T_4_. This is also the case for liquid and soft gel capsule formulations (101). Most data, especially sporadic case reports, focus on adults and there is a need to augment our knowledge about the effects of food on l-T_4_ in children. In the presence of problems of l-T_4_ absorption, switching from tablets to liquid l-T_4_ or soft gel capsules can in part solve the problem, and observing appropriate intervals between taking l-T_4_ and eating can also reduce the possibility of interaction (105).

#### 5.1.1 Infant Formula, Cow’s Milk, and Breast Milk

In newborns and infants with CH, there is also the possibility of interference from infant formula. There are few data in the literature but infant formula milk contains fat, proteins and lactose that can cause l-T_4_ to remain in the intestinal lumen which prevents it being absorbed (57). If patients are taking l-T_4_ with foods, they may need larger doses to maintain euthyroidism (55). One study by Chon et al. demonstrated that in adults, cow’s milk which is frequently ingested with breakfast can interfere with absorption (106). 1000 µg l-T_4_ in tablet form alone or together with 355 mL of 2% cow’s milk was administered to healthy adults. Peak serum TT_4_ concentrations and the area under the curve were reduced by nearly 8% in patients taking l-T_4_ with cow’s milk (106). It is possible that breast milk, which contains only 20-30% of the calcium in cow’s milk, is also decreases l-T_4_ bioavailability (55). However, no data are reported in the literature for children.

#### 5.1.2 Soy Based Formula

Some data, deriving mostly from old paediatric case reports, seems to show that soy infant formulas, as well as soy-containing baby foods, are related to altered thyroid function in early childhood (107–109).

Soy formula is often given to infants as an alternative to milk protein formula, especially in cases of intolerance to other formulas.

In 1959, Van Wyk et al. reported an infant fed with soy-based formula who presented cretinism and goitre at 10 months of age (108), which mostly resolved after the discontinuation of the soy-based diet. In another report on 78 patients under the age of one year, Pinchera et al. described the interaction between soya and l-T_4_ in a 3-week-old child fed on soy-based baby formula because of suspected lactose intolerance. Despite high doses of l-T_4_ (15 µg/kg per day), the infant’s TSH values remained elevated (248 mIU/L). Only after substituting the soy-based formula with another formula, did TSH decrease (110).

Since the 1960s soy formulas have been supplemented with iodine and it wasn’t until 1995 that other cases of soy-induced goitres in infants were reported (111, 112). Conrad et al. studied the influence of diet on thyroid function in paediatric CH patients, to investigate whether soy formula-based feeding can prolong the presence of increased TSH. The study included eight children on a soy formula diet and 70 on a non-soy diet. The children fed the soy formula had a more prolonged increase in TSH levels than children on non-soy formulas (113). The authors emphasized the need to frequently control levels of TSH in infants fed with soy formulas so that l-T_4_ dosage can be adjusted accordingly.

*In vitro* studies indicate that phytoestrogens may affect the synthesis of T_3_ and T_4_ by inhibiting thyroid peroxidise (114). However, in a meta-analysis of 14 studies, Messina and Redmond found no significant adverse clinical effects on thyroid function in healthy adults but the l-T_4_ dose might need to be increased in hypothyroid patients. For infants below the age of three years with CH (115), maximum attention appears necessary, given the thyroid-dependence of the central nervous system.

#### 5.1.3 Fruit Juices

Some data suggest that fruit juices - particularly grapefruit, orange, and apple - also interfere with l-T_4_ treatment by blocking the transporters carrying l-T_4_ from the small intestine into the blood (116, 117). Lilja et al. (118) described an adult hypothyroid female, previously treated with l-T_4_ with success, who presented a sudden new increase in TSH (more than 60 mU/L) with a reduced FT_4_ concentration (6.4 pmol/L) after consuming grapefruit juice. Her hormone levels returned to within the normal range only after the woman reduced her consumption of this juice. These results confirm the data of a randomized study of 10 healthy volunteers who drank grapefruit juice 1 h before taking l-T_4_,and presented a reduction of 9% in l-T_4_ absorption (118). Meyer et al. observed that THs upregulate expression of the transporter OATP2B1 hypothesising that hypothyroidism influences the interaction of juice and l -T_4_ (119). Recently Tesic et al. reported a 31-year-old female patient with persistent hypothyroidism despite treatment with high doses of l-T_4_ (alone or in combination with liothyronine) who had been taking l-T_4_ tablets with juice or mint tea. The patient was advised to take l-T_4_ with water, and her TSH levels normalized and her fT_4_ levels increased in a few days (120). Although the data are scarce and, at times, conflicting, it seems prudent not to take l-T_4_ with juice, but it does not seem necessary to eliminate fruit juice consumption completely.

Deiana et al. (121) reported a 37-year-old patient treated with l-T_4_ and with euthyroidism after thyroidectomy, who presented an unexpected TSH increase after eating large quantities of papaya (5-6 fruit a day). She was advised to stop eating the fruit completely and after 45 days her serum TSH concentration returned to within the normal range. Papain reduces gastric acid secretion which lowers l-T_4_ absorption; other components of the fruit, as well as fibre terpenoids, saponins, alkaloids, and flavonoids, may also reduce l-T_4_ absorption (121).

### 5.2 l-T4 – Drugs/Diseases Interaction

Many conditions or drugs may influence the gastric environment and affect the absorption of l-T_4_. Tablets of l-T_4_ need to be dissolved in an acid environment. Subjects with achlorhydria, reduced gastric acidity or who are taking PPIs do not have the ideal gastric environment to dissolve l-T_4_. PPIs are increasingly prescribed to children (122) and are effective treatments for many gastric diseases, duodenal ulcers, non-steroidal anti-inflammatory-induced ulcer-related prophylaxis, *Helicobacter pylori* in combination with other medications, gastro-esophageal reflux disease and eosinophilic esophagitis. They are also prescribed for functional dyspepsia, chronic cough, and infantile reflux (122).

In adult goitrous patients taking l-T_4,_ omeprazole assumption led to significant increases in serum TSH; this effect was reversed by increasing the dose of l-T_4_ or by discontinuing the use of omeprazole (123). Similar results have been reported for lansoprazole, but not pantoprazole or esomeprazole taken for one week (57). Antiacids also appear to interfere with thyroxine absorption by influencing gastric pH (57).

The recently developed liquid l-T_4,_ dissolved in an alcoholic solution, does not need an acid environment as it can be directly absorbed through the intestinal mucosa (124).

Besides drugs, pre-existing malabsorption diseases or disorders that impair gastric acidity also affect the bioavailability of l-T_4_ (125). There are some reports of elevated serum TSH levels in patients with coeliac disease and inflammatory bowel diseases taking doses of thyroxine that were previously sufficient to normalise serum levels (125).

Some studies report similar findings for patients with *Helicobacter pylori* infection and atrophic gastritis – both of also which impair gastric acidity (125).

Several reports regarding cases of l-T_4_ malabsorption in patients with various manifestations of CD have been reported (125, 126). For example, in a study conducted on seventy-nine children with permanent CH, 6 patients (4 girls, 2 boys) were positive for CD antibodies, showing a higher-than-normal prevalence of this disease in children with permanent CH. The available data show that a gluten-free diet results in the reduction of l-T_4_ dose requirements (127). Because the symptoms of some forms of CD are subtle, several authors suggest screening with CD markers in patients with hypothyroidism who need higher than normally expected doses of l-T_4_.

Franzese et al. report an infant with CH who developed, during l-T_4_ replacement therapy, a cow’s milk protein intolerance and subsequently CD. Both milk protein intolerance and CD affect the intestinal absorption of l-T_4_, making the management of CH difficult. After starting a diet with a hydrolyzed milk protein and maintaining the dose of l-T_4_ at 12 µg/kg dose, serum T_4_ improved and TSH decreased (128). A case of l-T_4_ malabsorption with lactose intolerance was reported in a 55-year-old woman with primary hypothyroidism whose TSH levels were persistently high (128).

After being diagnosed with oligo-symptomatic lactose intolerance, the patient was given a lactose-free formulation of l-T_4_ (150 mg daily) and began following a lactose-restricted diet; after 3 months the problem had resolved (129).

The presence of a pylori infection and atrophic gastritis of the body of the stomach, causing bacterial production of urease that neutralises gastric pH, may determine a decreased TSH suppression (123).

An infant with confirmed CH taking l-T_4_ experienced a possible drug interaction with simeticone (130) which is used to treat infant colic, a condition frequently seen by paediatricians. In this case, despite adequate l-T_4_ dosage, TSH was high, suggesting undertreatment. This case highlights the importance of regularly reviewing a patient’s clinical history and considering the possibility of drug interactions in unusual circumstances, especially where patients need high doses despite good compliance and proper administration (131). Clinicians should alert the parents of CH children starting l-T_4_ about the use of medicines containing simeticone (130).

Small intestinal bacterial overgrowth (SIBO) is a heterogeneous condition with nonspecific symptoms characterized by the presence or increase of atypical bacteria in the small intestine that unbalances intestinal microbiota. SIBO is characterized by symptoms such as flatulence, distension and abdominal pain. These symptoms might be indistinguishable from those presented by patients with functional gastrointestinal disorders. In most cases, SIBO is associated with motility or inflammatory disorders (132). The abnormally high levels of bacteria in the small intestine can cause malabsorption and suboptimal responses to narrow therapeutic index medication which are absorbed in the small intestine, such as l-T_4_ (133). In one study on patients with SIBO, l-T_4_ tablets and a compounded oral suspension were not absorbed efficiently, resulting in below optimal control of serum TSH. When patients switched to l-T_4_ sodium oral solution, TSH levels fell and symptoms resolved (133).

Malabsorption of l-T_4_ has also been described in patients infected with *Giardia lamblia*. Giardiasis is a common intestinal infection, but it has only rarely been reported as a cause of impaired l-T_4_ absorption (124). Giardiasis is probably under-diagnosed; in developing countries with poor sanitation its prevalence is about 20% while in industrialised countries prevalence ranges from 3% to 7% (124). Intestinal giardiasis can cause maldigestion, malabsorption and diarrhoea, leading to anaemia, weight loss and growth retardation (124). In cases of giardiasis infection in patients taking l-T_4_, switching from tablets to oral solutions revert decreased l-T_4_ absorption (124).

Short bowel syndrome (SBS), a major cause of intestinal failure in children, is diagnosed when the length of the small intestine is 25% shorter than expected for the child’s age (131). In paediatrics, the most frequent causes of SBS are resections secondary to necrotizing enterocolitis, gastroschisis, intestinal atresia and intestinal volvulus (134). SBS can cause a number of metabolic changes in the body, which can affect growth, intestinal adaptation and lead to metabolic bone disease as well as an impaired functioning of the hypothalamic-pituitary-thyroid (HPT) axis (135). Passos et al. reported six consecutive cases of children with SBS with associated hypothyroidism during the period of intestinal rehabilitation (135).

### 5.3 l-T4 – Dietary Fibre

A fibre-rich diet or dietary fibre supplements may also significantly reduce the bioavailability of l-T_4_ due to a non-specific link to fibre in the intestinal lumen causing malabsorption of the hormone (125). Products containing insoluble dietary fibre increase bowel motility, potentially altering the intestinal absorption of l-T_4_ (78). Although not all authors agree (135), in cases of dietary modifications which increase fibre intake, it may be necessary to monitor TSH levels and increase the dose of l-T_4_ (136).

### 5.4 l-T4 – Essential and Trace Elements

The possibility of interactions between levothyroxine and essential and trace elements has been investigated. Di- and trivalent elements, especially calcium and iron, appear to decrease l-T_4_ bioavailability (137). The mechanisms involved are not entirely clear but may connected to unspecific adsorption and the formation of insoluble complexes in the intestine (138). Parents should thus be informed about the possible adverse effects of calcium and iron supplementation.

#### 5.4.1 Calcium

CH may occur in association with congenital parathyroid hormone (PTH) insufficiency which leads to hypocalcaemia and low secretions of PTH (138). The combination of CH and PTH insufficiency occurs in patients with 22q11 microdeletion/DiGeorge syndrome, or due to PTH resistance in pseudohypoparathyroidism (139, 140). Metabolic bone disease is very frequent in preterm infants, occurring in 20-30% of very LBW infants (VLBW, <1500 g) and in 50-60% of extremely LBW infants (ELBW, <1000 g), who are at an increased risk for CH (141). The introduction of calcium-based supplements to improve PTH insufficiency could interfere with the absorption of l-T_4_.

In adults, calcium carbonate may reduce l-T_4_ absorption, resulting in a significative reduction of FT_4_, higher TSH levels in 20% of patients. These TH alterations were normalized after calcium carbonate discontinuation (142). There are several instances in the literature of interaction between calcium carbonate and l-T_4_ (143), and in a large observational study on patients assuming l-T_4_ tablets or iron supplements, in which the authors observed a significant increase of serum TSH in 4.4% of patients in the first group and 7.5% in the second group, concluding that supplementation with calcium and iron may cause a reduction in the absorption of l-T_4_ (62).

Other calcium preparations also potentially interact with l-T_4_. Diskin et al. (144) looked at TSH levels in more than 60 patients assuming l-T_4_ with different phosphate binders (mean dose 95–98 µg/day). The reported that only calcium carbonate, but not calcium acetate, resulted in significantly higher TSH levels. Zamfirescu et al. (145) studied the effect of calcium formulations (acetate, citrate, and carbonate) delivering a dose of 500 mg of elemental calcium on the absorption of l-T_4_ tablets at a dose of 1000 µg in eight healthy adults. For all the calcium preparations, taking the calcium supplement at the same time as the l-T_4_ tablets reduced absorption of l-T_4_ by 20 to 25%. The researchers stressed the importance of taking the examined calcium formulations and l-T_4_ at different times.

Morini et al. (146) studied a cohort of 50 postmenopausal women with hypothyroidism taking co- calcium supplements containing elemental calcium with a dose of 600-1000 mg for day. When the supplements were taken at the same time as or within 2 hours of l-T_4_ ingestion, the majority of patients presented significant increases in TSH levels, blood pressure, total cholesterol levels, and fasting glycemia.

Some data suggest that in adults the interaction of l-T_4_ and calcium can be reduced by replacing l-T_4_ tablets with liquid l-T_4_. Benvenga et al. reported data for 12 hypothyroid patients taking calcium carbonate (1000 mg/day) and assuming tablets or liquid l-T_4_ formulations, concluding that liquid l-T_4_ may be more resistant to sequestration by calcium (147). Mazokopakis et al. (148) found that only 8.4% of more than 150 patients were taking calcium carbonate at least 4 h before or after l-T_4_.

#### 5.4.2 Iron

Anaemia, defined as a haemoglobin levels of two standard deviations below the mean for age, is prevalent in infants and children worldwide with microcytic anaemia due to deficient iron intake being the most common type in children (149). Iron deficiency anaemia (IDA) can lead to cognitive problems that can be prevented or ameliorated with iron supplements or increased intake of iron in food (149). The phenolic, carboxylate, and amine functional groups on l-T_4_ molecules enable them to react with ferrous salts to form insoluble or only partially soluble complexes, which reduces the absorption of l-T_4_. Iron also plays an important part in TH synthesis and amino acid metabolism (150). Physiologically, IDA is able to impair THs metabolism, decrease T_4_ and T_3_ levels, reduce the peripheral conversion of T_4_ to T_3_ and T_3_ metabolism and decrease hepatic T4-5’-deiodinase leading to an increase in circulating TSH activity (151).

Anaemia is often present in infants affected by CH with severity depending on the degree of neonatal hypothyroidism which, if present during development, can lead to persistent health problems also after thyroid replacement therapy is started (152). “Uncomplicated” anaemia secondary to hypothyroidism responds to thyroid replacement therapy alone (153). Anaemia in hypothyroidism must be thoroughly evaluated because treatment must address its cause.

Gökdeniz et al. (154) showed that more than 15% of children with IDA presented concomitantly subclinical hypothyroidism, and Metwalley et al. (155) observed that primary school children with IDA were likely to develop SH and intellectual dysfunction. The presence of anaemia could influence the outcome of l-T_4_ therapy. Campbell et al. reported a lower efficacy of l-T_4_ treatment in adults when the drug was administered together with ferrous sulphate, with an increase in TSH levels in 79% of patients (156). A reduced absorption of l-T_4_ due to the co-administration of ferrous sulphate has been reported in a number of patients (157). Shakir et al. (158) suggest that interaction can occur even if patients maintain an interval of 4–6 h between taking l-T_4_ and supplements or medicines containing iron. Leger et al. report a 60-year-old female, previously treated with success with l-T_4_, who developed hypothyroidism after starting to take ferrous fumarate daily (TSH 243 mU/L, T_4_ <0.52 pmol/L). The woman’s thyroid function normalised 2 months after she stopped taking the iron supplement (159). Finally, Atruktsang et al. (160) recorded the time taken to achieve euthyroidism in over 600 subjects taking l-T_4_ as well as the number of dose adjustments that were necessary; among the patients whose dose need to be adjusted three or more times, a significant number used ferrous supplements.

Some data seem to suggest that, as with calcium, the use of an oral liquid form of l-T_4_ can help reduce problems of absorption correlated to iron supplementation. For example, in a small population of hypothyroid patients, Benvenga et al. (147) investigated l-T_4_ interaction with iron, observing a significant decrease in TSH levels in those who switched from tablets to oral liquid l-T_4_.

#### 5.4.3 Aluminium

Many antiacids contain aluminium. A number of case studies in adults report l-T_4_ malabsorption caused by the concomitant use of aluminium hydroxide (161). Liel et al. investigated five hypothyroid subjects treated with l-T_4_ who took gel tablets containing aluminium hydroxide for 2-4 weeks, observing a significant increase in serum TSH during the period of aluminium hydroxide ingestion (138).

#### 5.4.4 Iodine

Iodine is essential for THs which play an important role in foetal development and early infancy. Iodine deficiency in children may lead to reduced physical and intellectual capacity (162). In iodine-deficient areas, CH caused by iodine deficiency has a significant incidence (34). Preterm infants are at particular risk, also because parenteral nutrition and preterm infant formulas, including those used in hospital settings, do not always provide adequate iodine (163). Excess iodine intake may also cause a physiological decrease in THs synthesis, which is usually transient, due to the Wolff-Chaikoff effect (164). The thyroid’s ability to recover from the Wolff-Chaikoff effect is not fully mature until 36 to 40 weeks of gestation, meaning that preterm infants have a greater risk of prolonged hypothyroidism. Sources of excess iodine include iodine-containing antiseptics, radiographical contrast agents, and excess maternal intake of iodine (from diet or supplements) transmitted to infants in breastmilk (34).

The main dietary sources of iodine are iodized salt, seafood, and dairy products (165). Seaweeds are rich in iodine but the iodine content of seaweeds and dairy products varies considerably (166). Sea salt, Himalayan salt, and salt used in food processing is not always rich in iodine (148). Foods such as soybeans, cruciferous vegetables, and sweet potatoes contain substances capable of interfering with the thyroid’s uptake of iodine but in healthy individuals with adequate iodine intake, consumption of these foods does not lead to thyroid dysfunction (115). Vegans should use iodized salt and/or consume sea vegetables to minimise their risk of developing iodine deficiency (165).

The aetiology of hypothyroidism is multifactorial, but the most frequent causes are excess iodine intake and/or critical illness. Exposure to iodinated Contrast Media (iCM) is an increasingly common source of excess iodine in vulnerable patients (166). The thyroid gland has two auto-release mechanisms to manage high intra-thyroid iodine doses, namely the *Via* the Wolff–Chaikoff effect and the escape phenomenon (167). However, in newborns and infants these mechanisms are not mature making them prone to iCM toxicity (168). Infants with iCM toxicity typically have low FT_3_ plasma concentrations with low or normal FT_4_ and low or normal TSH, consistent with a non-thyroidal disease syndrome.

#### 5.4.5 Selenium

The enzymes, iodothyronine deiodinases, glutathione peroxidases, and thioredoxin reductases, involved in TH biosynthesis and metabolism, regulation of the redox state, and protecting the thyroid from oxidative damage are selenoproteins. Thus, low selenium levels may lead to hypothyroidism: see below in the section related to parenteral nutrition (169).

### 5.5 l-T_4_ – Vitamin Interaction

It has been shown that increased gastric pH can influence the absorption of l-T_4_. Jubiz et al. studied the effects of vitamin C, capable of lowering gastric pH, on l-T_4_ absorption (170) They conducted a study in 31 hypothyroid subjects with gastritis under l-T_4_ therapy, taking a median dose of 100 µg in tablet form, with 120 mL of water with or without 500 mg vitamin C in solution. While on vitamin C, TSH levels fell in all patients, and FT_3_ and FT_4_ levels increased significantly. Antúnez et al. (171) observed similar results in 28 patients with elevated TSH levels, despite being on a l-T_4_ dose higher than 1.70 µg/kg. Patients were asked to take l-T_4_ tablets with 1 g of vitamin C (effervescent tablets, dissolved in 200 mL of water) for 6–8 weeks. Significant decreases in serum TSH levels (from 9.01 ± 5.51 mU/L to 2.27 ± 1.61 mU/L) were achieved.

Biotin is a vitamin commonly used in multivitamin preparations and used to treat progressive multiple sclerosis, several inherited metabolic diseases and acquired dermatologic diseases. It can interfere with many endocrine laboratory assays (including TSH and FT_4_) (172), which can lead to erroneous or delayed diagnosis of CH. There are cases in the literature of children taking high doses of biotin as therapy for metabolic disease being wrongly diagnosed with hyperthyroidism (172).

The correct assessment of thyroid function may be influenced by biotin therapy (173). High levels of biotin–streptavidin can compete with biotinylated components leading to inaccurate and misleading results characterised by increased FT_3_, FT_4_, levels and a reduction in TSH levels, which may lead to misplaced suspicion of Graves’ disease (173). This could lead to erroneous therapeutic strategies with serious consequences in the first three years of life given the thyroid dependency of the central nervous system in this period.

Wijeratne et al. conclude that interference peaks at approximately 2 h after biotin ingestion and can persist for up to 24 h (174). In a newborn with trisomy 21, CH and partial biotinidase deficiency, because of interference with the TSH assay from concurrent biotin administration routine screening detected “normal” TSH levels leading to a delayed diagnosis of CH (172).

### 5.6 l-T_4_ – Nutrition Route

In paediatrics, the most basic and important method of nutritional intervention is enteral nutrition (EN) (175). EN is introduced for patients who cannot meet their energy and nutritional needs through normal feeding. EN is often required for children with growth retardation, inadequate weight gain, or weight deficit, and is employed in the treatment of diseases such as Crohn’s, and food allergies or intolerance (175). In neonates, EN is required in situations such as premature or necrotizing enterocolitis (175).

Reis et al. (176) carried a multicentre study in Brazil to evaluate possible drug-EN interactions. The authors found l-T_4_-EN interaction to be one of the most frequent and clinically significant interactions. In an earlier study, Dickerson et al. (177) evaluated 13 hypothyroid patients in hospital: all participants received EN; their l-T_4_ doses were kept the same as they were before the patients were hospitalised for 20 ± 5 days. Eight of the thirteen patients developed subclinical or overt hypothyroidism, indicating that it is vital to monitor hypothyroid patients who receive continuous EN and who are being treated with l-T_4_. Manessi et al. (178) discovered that l-T_4_, may be adsorbed by enteral feeding tubes and hypothesised that this mechanism was related to a reduction in treatment efficacy. However, Wohlt et al. (179) concluded that the amount of l-T_4_ absorbed by feeding tubes is likely to be clinically irrelevant, and that impaired l-T_4_ absorption may be caused by the concomitant ingestion of food. Pirola et al. (180) studied 20 euthyroid patients, a day after surgical invention, to compare the efficacy of different formulations of l-T_4_ administered while patients were attached to an enteral feeding tube. EN was stopped for 30 min before and after powdered l-T_4_ tablets were administered, whereas liquid l-T_4_ was administered *via* the feeding tube without interrupting EN. There were no significant differences in the results obtained by the two methods, leading the authors to conclude that EN does not impact the absorption of liquid l-T_4_.

Parenteral Nutrition (PN) is used when normal and enteral feeding are impossible (181). Some reports show that long-term PN is a risk factor for low iodine and/or selenium levels, causing hypothyroidism, which in some cases is severe, stressing the importance of dosing of these elements and evaluating THs (182, 183).

## 6 Effects on Thyrotropin Releasing Hormone (TRH) and TSH Levels

Drugs and medications frequently influence thyroid function altering the secretion of TRH or TSH (184), even if only a small subgroup (glucocorticoids or GCs, dopamine agonists or DAs, etc) affects the hypothalamus or pituitary (100). **Tables 1** and **2** report the drugs and medications which affect thyroid function in children with intact an hypothalamus-pituitary-thyroid axis (**Table 1**) and in those dependent on exogenous l-T_4_ (**Table 2**), also describing the mechanisms of action.

**Table 1:** Medical conditions, drugs and medications potentially affecting l-T_4_ absorption in children.

Diseases	Drugs and medications
Coeliac disease	Acid suppression therapies (proton pump inhibitors, H2 receptor antagonists, sucralfate)
Lactose intolerance	Bile acid sequestrants (cholestyramine, colestipol, and colesevelam)
Atrophic gastritis	Calcium salts (carbonate, citrate, and acetate)
*Helicobacter pylori*	Ferrous sulphate
Chronic cholestasis and liver cirrhosis	Multivitamins (containing ferrous sulfate or calcium carbonate)
Short bowel disease	Cation exchange resins (Kayexelate)
Inflammatory bowel disease	Simethicone
Intestinal surgery	Ciprofloxacin
Giardiasis	β-blockers
Small intestinal bacterial overgrowth (SIBO)	Charcoal
**Foods**	
Ingestion with a meal (food intake)	Oral bisphosphonates
Cow’s milk?	Phosphate binders (Sevelamer hydrochloride, aluminium hydroxide)
Dietary fibre	Rifampicin
Grapefruit	Polystyrene sulfonate
Soybeans and soy	Tricyclic antidepressant
Papaya	

**Table 2:** Main drugs potentially affecting thyroid function or thyroid hormone levels in patients with congenital hypothyroidism.

Drugs	More frequent use in children	Effects
Iron	Anaemia	Inhibition of l-T_4_ absorption
Calcium	Hypocalcaemia	Inhibition of l-T_4_ absorption
Aluminum/magnesium hydroxide	Gastro-esophageal reflux (antiacid)	Inhibition of l-T_4_ absorption
Sucralfate	Gastro-esophageal reflux (antiacid), gastritis, ulcers	Inhibition of l-T_4_ absorption
Colestyramine	Chronic cholestasis/inherited hyperlipidemia	Inhibition of l-T_4_ absorption
Colestipol	Chronic cholestasis/inherited hyperlipidemia	Inhibition of l-T_4_ absorption
Carbemazepine/Oxcarbemazepine	Seizures, mood disorders	TSH suppression; Increased hepatic metabolism
Glucocorticoids	Acute respiratory conditions as asthma and croups, inflammatory bowel diseases, nephrotic syndrome, etc	TSH suppression; inhibition of 5’ deiodinase; decreased thyroxine binding globulin levels
Nonsteroidal anti-inflammatory medications	Fever, musculoskeletal pain	Displacement from thyroxine binding globulin (laboratory artifact)
Phenytoin	Seizures	Increased hepatic metabolism; displacement from thyroxine binding globulin (laboratory artifact)
Phenobarbital	Seizures	Increased hepatic metabolism
Lithium	Acute mania and bipolar disorder	Inhibition of T4/T3 secretion
Dopamine agonists	Hyperprolactinemia	TSH suppression
Propranolol	Cardiac dysrhythmias	Inhibition of 5’ deiodinase
Rifampicin	Treatment of several types of bacterial infections	Increased hepatic metabolism
Furosemide	Renal, cardiac and peripheral oedema, ascites, hypertension)	Displacement from thyroxine binding globulin (laboratory artifact)
Iodide	Drugs containing iodide	Inhibition of 5’ deiodinase
Amiodarone	Cardiac arrhythmias	Inhibition of 5’ deiodinase, Thyroiditis
Nicotinic acid	Paediatric lipid disorders?	Decreased thyroxine binding globulin levels
Methadone	Life-Limiting Illness?	Increased thyroxine binding globulin levels

Generally, the widely used GCs are not able to induce clinical central hypothyroidism even when used for long periods at high doses. DAs do not typically lead to clinically evident central hypothyroidism, but in patients with nonthyroidal illness they may cause an additional TSH suppression, which could potentially result in iatrogenic central hypothyroidism. Rexinoids, in contrast, frequently induce clinically relevant central hypothyroidism in the majority of subjects, who will require l-T_4_ treatment and follow up to control serum FT_4_. This new type of drug is likely to be used on increasing numbers of patients (advanced cancer, metabolic disorders, dermatologic disorders), and it is therefore important to consider the risks of possible side-effects so that they can be managed with treatment.

l-T_4_ is also the first line treatment for secondary hypothyroidism, which in developing countries is frequently caused by CH, Hashimoto thyroiditis, thyroidectomy, or iodine deficiency (185). The interference of food on l-T_4_ absorption, as previously mentioned, is known to affect and the efficacy, and in some cases, safety of therapy but there is a lack of awareness among patients and health care workers about this potential problem.

### 6.1 Glucocorticoids

Glucocorticoids are often used in paediatrics for their immunosuppressant and anti-inflammatory properties, individually or in combination with other drugs both short and long term (186, 187). It is well known that GCs affect serum TSH levels in animals and humans, reducing TSH secretion by directing effecting TRH a hypothalamic level (188). Physiologically, it appears that levels of hydrocortisone have a key role in the diurnal variation of serum TSH, with lower levels in the morning and higher levels at night (189, 190). Some data have shown that high dose GCs may suppress serum TSH in normal subjects and also in hypothyroid patients (191). Nevertheless, long-term high doses of GCs or the cortisol excess typical of Cushing’s syndrome do not seem to result in clinical central hypothyroidism necessitating l-T_4_ treatment (192). Dexamethasone doses as low as 0.5 mg or prednisone 30 mg can reduce TSH levels significantly (192).

This suppressive effect of GCs on TSH secretion appears to be controlled by the hypothalamus through the inhibition of TRH (193). In humans, GCs receptors are present in the TRH neurons of the paraventricular nucleus and high dose GCs can reduce TRH mRNA levels in the hypothalamus, acting on the TRH gene, probably the primary mechanism for lowering TSH secretion from the hypophysis (194).

The effect of GCs on TSH should be taken into consideration when checking thyroid function in CH patients, in order not to mistake an overtreated condition with a physiological response to GC treatment.

### 6.2 Dopamine

Dopamine is widely used in critical illnesses to increase blood pressure, cardiac output, urine output, and peripheral perfusion in neonates, infants, and older children with shock and cardiac failure (195).

Dopamine and dopamine agonists can suppress serum TSH and affect the activation of dopamine D2 receptors, reducing TSH pulse amplitude without significantly altering TSH pulse frequency (196, 197). Interestingly, in rats, dopamine stimulates hypothalamic TRH release, but because its overall effect is to lower serum TSH, the inhibitory effect on the pituitary exceeds the first effect (198).

Data on neonates with nonthyroidal illness (NTI) syndrome treated with dopamine infusions indicate that dopamine and NTI have an additive effect on suppressing the HPT axis, placing these individuals at risk of iatrogenic central hypothyroidism (199, 200). It is not clear whether treatment with l-T_4_ should be given to patients with NTI who are receiving dopamine infusions but, given the thyroid dependence of the central nervous system, the risks of not treating children with CH under 3 years of age should be carefully considered.

### 6.3 Antiepileptic Drugs

Several drugs used to treat epilepsy such as carbemazepine (CBZ), oxcarbemazepine and valproic acid (VPA) could increase the metabolism of TH through the hepatic P450 system and could also impede pituitary responsiveness to hormonal feedback and cause central hypothyroidism (201, 202). However, several investigations suggest that the hypothalamic-pituitary axis is unaffected by these drugs and a specific mechanism has not been discovered (203). Effects appear to be different in relation to the medications considered. For example, in a study evaluating the use of VPA, CBZ and levetiracetam (LEV), thyroid dysfunction was frequent in children taking VPA or CBZ as a monotherapy, but absent in those taking LEV. The authors suggest that, in children with a predisposition for thyroid disease, LEV should be considered over VPA and CBZ, if appropriate for seizure and epilepsy type (204).

## 7 Unresolved Questions and Conclusions

Various factors, both exogenous and endogenous, can influence and modify the kinetics of the absorption of l-T_4_. Pending other targeted studies in the paediatric population, the timing of food intake in relation to l-T_4_ administration should always be considered as having the potential to interfere with absorption and parents and other caregivers should be advised to delay the consumption of any food for at least 30, and preferably 60 minutes, after taking l-T_4_.

Caregivers, and also healthcare personnel dealing with CH patients, must be informed about possible interactions with food, drugs and the possible influence of diseases on thyroid function and l-T_4_ absorption, in order to minimise the negative effects of poorly conducted treatment on the function and development of the central nervous system, especially in the first three years of life.

It is well established that conditions causing malabsorption or reduced gastric acidity can impair the absorption of l-T_4_ and the sudden onset of apparently resistant hypothyroidism in patients treated with l-T_4_ may be the only clinically observable feature of some of these disorders.

Drugs may interact with TH levels in patients with CH through several mechanisms, influencing thyroid status at the hypothalamic, pituitary, or thyroid level, or affecting the binding of THs to protein carriers and the conversion of T_4_ to T_3_, as well as the final metabolism and recycling of TH. These drugs may also affect the efficiency of exogenous l-T_4_ treatment, requiring vigilance to prevent both undertreatment and overtreatment and to interpret laboratory findings correctly to avoid inappropriate therapeutic changes.

Data about the interference of different foods on l-T_4_ absorption in children are very scarce and therefore attention is needed. Liquid formulations are better able to resist interference but all the same, parents and health professionals must remember not to administer l-T_4_ therapy too close to food.

## 8 Main Concerns

1. CH is one of the most common preventable causes of ID.
2. Newborn CH screening allows early diagnosis and treatment, significantly reducing the risk of irreversible ID and neurologic damage.
3. Clinicians should be aware that certain forms of cCH or milder forms of primary CH may be missed by newborn screening, and it is therefore fundamental that healthcare works are able to recognise the clinical signs and symptoms of CH in order to avoid erroneous or delayed diagnoses.
4. Prompt diagnosis and treatment with adequate doses of l-T_4_ leads to excellent neurodevelopmental outcomes in most patients with CH. However, all patients should be closely followed after starting and while under treatment, to monitor their neurological and psychological development.
5. The failure to adequately control CH with oral l-T_4_ is a frequent clinical problem.
6. Before increasing the l-T_4_ dose in a patient with CH previously well-controlled, it is mandatory to assess adhesion to treatment.
7. In order to avoid the risk of suboptimal care and patient management, it is essential that caregivers and healthcare personnel are aware of the potential effects of food, drugs and diseases on thyroid function and l-T_4_ absorption. Possible interferences with l-T_4_ treatment, should be re-evaluated at each follow-up visit.
8. l-T_4_ oral solution may have a better absorptive profile than tablets and switching from tablet to liquid l-T_4_ should be tried before increasing the dose of l-T_4_.
9. The risk of overtreatment in the first months of life, and especially in the first 4 weeks, makes it fundamental that patients in this age group receive careful follow-up.

## Author Contributions

All authors participated in the writing of the manuscript and read and approved the final manuscript.

## Conflict of Interest

The authors declare that the research was conducted in the absence of any commercial or financial relationships that could be construed as a potential conflict of interest.

## Publisher’s Note

All claims expressed in this article are solely those of the authors and do not necessarily represent those of their affiliated organizations, or those of the publisher, the editors and the reviewers. Any product that may be evaluated in this article, or claim that may be made by its manufacturer, is not guaranteed or endorsed by the publisher.
